# Yixiao Formula Suppresses Myocardial Fibrosis Through UpregulatingmiR‐133a and Downregulating TGF‐*β*/Smads Signal Molecules

**DOI:** 10.1155/jdr/5533249

**Published:** 2026-01-21

**Authors:** Qiyao Zhao, Yalu Wen, Honghui Wu, Jiaoyue Li, Yunpeng Luo, Ping Li, Ye Zhang, Chaoyue Hu, Jukai Huang, Li Zhang, Xiaohui Yang

**Affiliations:** ^1^ Department of Nephrology and Endocrine (II), Dongzhimen Hospital, Beijing University of Chinese Medicine, Beijing, China, bucm.edu.cn; ^2^ Specialized Department for the Integrated Treatment of Diabetes and Obesity With Traditional Chinese and Western Medicine, Tongde Hospital of Zhejiang Province, Hangzhou, China, zjtongde.com; ^3^ Department of Respiratory Medicine, Beijing Hepingli Hospital, Beijing, China; ^4^ Department of Internal Medicine, Jingkai District, Dongfang Hospital, Beijing University of Chinese Medicine, Beijing, China, bucm.edu.cn; ^5^ Department of Scientific Research, Dongfang Hospital, Beijing University of Chinese Medicine, Beijing, China, bucm.edu.cn

**Keywords:** cardiac fibroblasts, diabetic cardiomyopathy, miR133a, TGF-*β*/Smads, traditional Chinese medicine

## Abstract

**Background:**

Yixiao formula (YXF), a traditional Chinese herbal medicine, has demonstrated clinical efficacy in alleviating symptoms of diabetic cardiomyopathy (DCM). The therapeutic mechanism underlying YXF′s effects on DCM remains poorly understood. Myocardial fibrosis is a key pathogenic mechanism in DCM, and previous studies have indicated that miR‐133a may be involved in its progression. Given that the TGF‐*β*/Smads signaling pathway is a well‐established mediator of myocardial fibrosis, investigating the mechanistic role of YXF through miR‐133a and the TGF‐*β*/Smads pathway warrants further exploration.

**Objective:**

The main objective of this study is to investigate the potential contribution of the TGF‐*β*/Smads pathway to the effects of YXF, as well as the role of miR133a, through in vivo DCM models and in vitro experiments.

**Materials and Methods:**

Spontaneously diabetic KKAy mice were used to establish a DCM model by continuous high‐fat feeding, with C57BL/6 mice as controls. Echocardiography, body weight, and blood glucose data were collected every 4 weeks to examine the effects of YXF on blood glucose levels and changes in cardiac function and structure in DCM mice. Immunohistochemistry, RT‐qPCR, and western blot were used to detect the expression levels of the TGF‐*β*/Smads pathway. Additionally, the potential molecular mechanism of YXF in mouse cardiac fibroblasts (MCFs) was investigated by knocking down miR‐133a.

**Results:**

YXF improved fasting blood glucose levels in DCM mice, promoted cardiac diastolic function, upregulated miR133a, and inhibited the expression of the TGF‐*β*/Smads pathway. Furthermore, when miR133a inhibitors were transfected under YXF intervention, we found that YXF′s inhibitory effect on the TGF‐*β*/Smads pathway was weakened.

**Conclusion:**

Through this study, we found that YXF can increase miR133a and inhibit the expression of the TGF‐*β*/Smads pathway, thereby inhibiting myocardial fibrosis and exerting a protective effect on DCM.

## 1. Introduction

Diabetes mellitus, primarily characterized by chronic hyperglycemia, can lead to various complications involving the heart, kidneys, nerves, and other tissues and organs. Its incidence rate has been increasing year by year, and it is predicted that by 2045, the global prevalence of diabetes among adults will reach 12.2% [1]. Cardiovascular disease is the leading cause of death among patients with Type 2 diabetes [2]. Diabetic cardiomyopathy (DCM), a common complication of diabetes, is often seen in patients with a disease duration of 5 years or more. Its clinical manifestations include changes in cardiac structure and diastolic and systolic functions, which ultimately lead to heart failure and endanger life safety [3]. The pathological mechanism of DCM is complex, with hyperglycemia being the key initiating factor. Oxidative stress, autophagy, inflammation, and myocardial fibrosis are all involved in the occurrence and development of DCM, among which myocardial fibrosis is the main pathological feature [4, 5]. Myocardial fibroblasts (MFs) are pathological cells that play an important role in promoting fibrosis [6]. Studies have shown that angiotensin II (AngII) can induce MF proliferation and promote collagen fiber deposition, thereby mediating myocardial fibrosis [7]. The TGF‐*β*/Smads pathway is a classical pathway of fibrosis. When activated, TGF‐*β*1 binds to receptors, further activating downstream phosphorylation of Smad2 and Smad3, which then transfer the TGF‐*β*1 stimulus signal to the nucleus to promote fibroblast proliferation and migration [8].

Currently, the treatment options for DCM are relatively limited. Traditional Chinese medicine (TCM) formulas and proprietary Chinese medicines have shown advantages in the treatment of DCM due to their multichannel, multitarget approach and mild side effects. A significant amount of basic and clinical research by TCM scholars on this disease has revealed that Chinese medicines can treat DCM through various physiological and pathological mechanisms, including antioxidant and anti‐inflammatory effects [9, 10]. YXF is a TCM formula used for the prevention and treatment of DCM. According to TCM theory, DCM is believed to result from “microblood stasis” in the heart, due to a deficiency of both qi and yin. The herbal components of YXF are designed to nourish qi and yin, promote blood circulation, and remove blood stasis. In clinical practice, YXF has been found to regulate glucose and lipid metabolism and improve symptoms in DCM patients. Previous studies have shown that YXF can interfere with renin–angiotensin system activation, reduce AngII levels, and exert cardioprotective effects [11].

MicroRNAs (miRNAs) are single‐stranded noncoding RNAs approximately 18–25 nucleotides long that bind to the RNA‐induced silencing complex (RISC) and repress target mRNA translation [12]. Differential expression of miRNAs has been reported in DCM [13]. Studies have indicated a significant downregulation of miR‐133a in the hearts of diabetic mice [14]. Based on this, the hypothesis that YXF alleviates myocardial fibrosis in DCM by upregulating miR‐133a and inhibiting the TGF‐*β*/Smads signaling pathway deserves further investigation. In this study, we explored the effects of YXF‐containing serum on high glucose‐induced mouse cardiac fibroblasts (MCFs), providing an in‐depth analysis of YXF′s pharmacological mechanism in DCM and experimental evidence for its therapeutic use in treating DCM.

## 2. Methods and Materials

### 2.1. Animals

The SPF‐grade C57BL/6J mice and KKAy mice used in the experiment were purchased from Beijing Huafukang Bioscience Co., Inc., with a Production License Number SYXK (Jing) 2019‐0008. The animals were housed in the SPF‐grade laboratory animal room at the Children′s Hospital affiliated with the Capital Institute of Pediatrics, with License Number SYXK (Jing) 2023‐0010. Both regular maintenance feed and high‐fat feed were purchased from Beijing Huafukang Bioscience Co., Inc. The animal experiment has been approved by the Animal Ethics Committee (Approval No. VS2126A01208).

### 2.2. Preparation of YXF

YXF consists of 10 Chinese herbs as follows: *P. heterophylla* (30 g), *Astragali Radix* (10 g), *Ophiopogon japonicus* (10 g), *Schisandra chinensis* (10 g), *Salvia miltiorrhiza Bunge* (10 g), *Angelica sinensis* (10 g), *Radix Paeoniae* (10 g), *Euonymus alatus* (10 g), *Fritillaria thunbergii* (10 g), and *Prunella vulgaris* (10 g). A total of 120 g raw herbal pieces, as above, were immersed in a 10‐fold volume of distilled water for 30 min and boiled for 1 h. Then, repeat this extraction procedure with the residue from the first extraction. Finally, the filtered solutions were combined and concentrated to a relative density of 0.77 g/mL (1 mL of extracted solution contains 0.77 g of raw herb extract). After cooling at room temperature, the liquid was separated and stored at 4°C. The raw herbal pieces were purchased from Dongzhimen Hospital, Beijing University of Chinese Medicine.

### 2.3. Drugs and Chemical Reagents

Losartan potassium tablets (National Medical Product Approval Number J20171081) were purchased from Dongzhimen Hospital of Beijing University of Chinese Medicine. Antibodies TGF‐*β*1 (Cat#ab215715), Smad2 (Cat#ab40855), and Smad3 (Cat#ab40854) were obtained from Abcam (Cambridge, United States); the antibody GAPDH (Cat#5174) was from Cell Signaling Technology (Danvers, United States); Goat Anti‐Rabbit IgG H&L (HRP) was from Abcam (ab6721); Immunohistochemical reagents were purchased from ZSGB‐BIO (Beijing, China): Rabbit two‐step detection kit (rabbit enhanced polymer detection system) (Cat#PV‐9001) and DAB chromogenic kit (ZLI‐9017). The animal total protein extraction kit (Cat#C510003‐0050) was purchased from Sangon Biotech (Shanghai, China). The RNA extraction kit (Cat#R021‐100) was from Beijing GeneBetter Biotechnology Co., Ltd. (Beijing, China), the reverse transcription kit (Cat#AT301) was from Beijing TransScript Biotech Co., Ltd. (Beijing, China), and the RT‐qPCR kit (Cat#G891) was from Applied Biological Materials Inc. (Richmond, Canada). DMEM high‐glucose medium (Cat#SH30022.01B) and penicillin–streptomycin (Cat#SV30010.01B) were obtained from Hyclone (Logan, United States); DMEM low‐glucose medium (Cat#CM10014) was from Maichen Technology (Beijing) Co., Ltd. (Beijing, China); Trypsin‐0.25% EDTA digestive juice (Cat#25200056) and fetal bovine serum (FBS) (Cat#F001‐106) were from Thermo Fisher Scientific Inc. (Waltham, United States); the ECL kit (Cat#SQ201L) was purchased from Shanghai Yamei Biotechnology Co., Ltd. (Shanghai, China). The transfection reagent (Cat#C0533‐0.5 mL) was from Shanghai Beyotime Biotechnology Co., Ltd. (Shanghai, China). miR‐133a inhibitor and scrambled miRNA were designed by GenePharma CO., Ltd. (Suzhou, China).

### 2.4. Model Establishment and Grouping

Then, 12‐week‐old male spontaneous diabetic KKAy mice and male C57BL/6J mice were kept under specific pathogen‐free conditions in the animal room of Capital Institute of Pediatrics (Beijing, China). The mice were reared in a constant temperature of 22°C and humidity of 55*%* ± 5*%*, with a 12‐h light cycle. KKAy mice were given a high‐fat diet, while C57BL/6J mice received a regular diet. After 2 weeks of acclimatization, blood samples were collected from the tail vein to measure fasting blood glucose (FBG), and a cardiac ultrasound was performed to assess diastolic function. Mice whose FBG level exceeds 13.9 mmol/L were considered diabetic, while those with diastolic dysfunction shown by cardiac ultrasound are classified as models of DCM.

The mice were randomly divided into four groups: (1) control group (untreated C57BL/6J mice, *n* = 24); (2) DCM group (untreated KKAy mice, *n* = 24); (3) DCM + YXF group (KKAy mice treated with 7.7 g/kg YXF daily, *n* = 24); and (4) DCM + losartan group (KKAy mice treated with 10 mg/kg losartan daily, *n* = 24). Mice in the DCM + YXF and DCM + losartan groups were intragastrically administered drugs at 0.1 mL/10 g daily for 12 weeks. In contrast, the control and model groups received the equivalent volume of distilled water in the same manner. Every 4 weeks (0, 4, 8, and 12 weeks), six mice of each group were randomly selected to record echocardiography and monitor blood glucose. Then, the heart tissues of each mouse were removed, fixed in 4% paraformaldehyde for histopathological analysis, and stored at −80°C for molecular biological analysis.

### 2.5. Ultra‐Performance Liquid Chromatography–Mass Spectrometry Analysis (UPLC‐MS/MS)

Chemical constituents in YXF‐medicated serum were analyzed by a UHPLC‐MS/MS system (UPLC, ExionLC AD, http://sciex.com.cn/) and a tandem mass spectrometry system (https://sciex.com.cn/). Chromatographic separation was performed on an Agilent SB‐C18 column (1.8 *μ*m, 2.1 × 100 mm) maintained at 40°C. The mobile phase consisted of ultrapure water (phase A) and acetonitrile (phase B) with the following gradient elution program: Phase B was linearly increased from 5% to 95% over 0–9 min, rapidly reduced to the initial 5% at 11 min, and then held until 14 min for column equilibration. The flow rate was set at 0.35 mL/min, and the injection volume was 2 *μ*L.

### 2.6. Oral Glucose Tolerance Test (OGTT)

Every 4 weeks, after fasting for 12 h, six mice were randomly selected from each group and treated with 2 g/kg glucose by gavage. Tail‐tip blood glucose was measured at 0, 30, 60, 90, and 120 min using a blood glucose meter.

### 2.7. Echocardiography

Transthoracic echocardiography was performed on lightly anesthetized, unconscious mice to evaluate cardiac function. Two‐dimensional, M‐mode, and tissue Doppler echocardiographic images were recorded using a VEVO 2100 imaging system (FUJIFILM, VisualSonics Inc.). Left ventricular ejection fraction (LVEF%) was measured from the parasternal short‐axis view at the aortic valve level. The *E*/*e* ^′^ ratio was calculated by pulse wave Doppler and tissue Doppler imaging to assess diastolic function. Left ventricular anterior wall thickness (LVAWT), left ventricular end‐diastolic diameter (LVIDD), and left ventricular diastolic posterior wall thickness (LVPWd) were measured from the parasternal short‐axis view at the aortic valve level. The investigator who performed the echocardiography was blinded to the treatments.

### 2.8. Histopathological and Immunohistochemical Study

The left ventricles of mice were collected and fixed in 4% paraformaldehyde for 48 h at 4°C. Then, the fixed tissue samples were dehydrated, embedded in paraffin, and sectioned into 4‐*μ*m slices. The slices were stained with hematoxylin and eosin (HE) and Masson′s trichrome to exhibit cardiac structure. The collagen volume fraction (CVF) and perivascular collagen area/luminal area (PVCA/LA) were measured with Image‐Pro Plus software. Immunohistochemistry was also used to evaluate fibrosis. Briefly, heat‐mediated antigen retrieval was performed, followed by blocking with 5% BSA in PBS, and then incubated with anticollagen I (1:750) and anticollagen III (1:2000) at 4°C overnight. After that, sections were incubated with HRP‐conjugated secondary antibody at 37°C for 30 min, followed by DAB staining.

### 2.9. Cell Culture

MCF cells (Cat#BNCC100314) were purchased from the Engineering and Technology Research Center of Industrial Microorganisms in Henan Province, China (Henan, China). Complete medium was prepared with 90% DMEM, 10% FBS, and 100 U/mL penicillin and 100 *μ*g/mL streptomycin. The MCFs were cultured in a 5% CO_2_ incubator at 37°C.

### 2.10. Preparation of YXF‐Containing Serum

Then, 20 SD rats were randomly divided into a blank group, a YXF group, and a losartan group after 3 days of adaptive feeding. The YXF group was administered 7.7 g/kg of YXF via gavage, while the losartan group received losartan via gavage. The blank group was given the same volume of distilled water. Daily oral administration continued for 7 consecutive days, followed by blood collection from the abdominal aorta 1 h after the final treatment. The whole blood was allowed to stand for 30 min, followed by centrifugation at 3000 rpm for 10 min to obtain the serum. The serum was then filtered and sterilized using a 0.22‐*μ*m filter and stored in separate containers at −80°C for future use.

### 2.11. Cell Transfection and Treatment

Cell transfection was conducted using the lipofection method. Before transfecting with the corresponding miR‐133a inhibitor or scrambled miRNA, MCFs were seeded into 6‐cm dishes. Transfection was performed when the cells reached 70%–80% confluence, according to the manufacturer′s instructions for the lipo8000 reagent. The transfection duration was 24 h, and the intervention concentration and time for the medicinal serum were 20% and 24 h, respectively.

The experimental groups were divided into six categories:

1. Control group: DMEM low‐glucose medium + blank serum
2. Model group: DMEM high‐glucose medium + blank serum
3. Losartan group: DMEM high‐glucose medium + losartan serum
4. YXF group: DMEM high‐glucose medium + YXF serum
5. miR133a inhibitor group: DMEM high‐glucose medium + YXF serum + transfection with miR133a inhibitor
6. miR133a NC group: DMEM high‐glucose medium + YXF serum + transfection with miR133a NC

### 2.12. Quantitative Real‐Time PCR

Cells from each group were seeded into 12‐well plates. After the intervention, cells were collected, and RNA was extracted using the RNeasy Mini Kit (Qiagen) according to the manufacturer′s instructions. RNA concentration and quality were determined using NanoDrop 2000. Following the reverse transcription kit protocol, the samples were incubated in a PCR machine at 42°C for 17 min and then at 85°C for 5 s to obtain cDNA, which was stored at −20°C for later use. The specific primer sequences are listed in Table 1. Samples and primers were diluted according to the PCR kit instructions, and the PCR system was set up. CT values were obtained using the machine, and the relative expression levels of the target genes were calculated using the 2^-*ΔΔ*Ct^ method.

**Table 1:** Primer sequences.

**Gene**	**Forward primer (3** ^′^ **-5** ^′^ **)**	**Reverse primer(5** ^′^ **-3** ^′^ **)**
TGF‐*β*1	CCACCTGCAAGACCATCGAC	CTGGCG AGCCTTAGTTTGGAC
Smad2	CTCTCCAACGTTAACCGAATG	CACCTATGTAATACAAGCGCAC
Smad3	CTGGCTACCTGAGTGAAG ATG	AGTAGGTGACTGGCTGTAGG
GAPDH	AGAAGGTGGTGAAGCAGGCATC	CGAAGGTGGAAGAGTGGGAGTTG
miR‐133a	—	TTTGGTCCCCTTCAACCAGCTG
U6	CTCGCTTCGGCAGCACATATACT	ACGCTTCACGAATTTGCGTGTC

### 2.13. Western Blot Assay

Cells from each group were seeded into 6‐cm dishes and collected after intervention. Total cellular proteins were extracted using a total protein extraction kit, and protein concentrations were determined using a BCA assay kit, with a uniform concentration of 20 *μ*g/20 *μ*L. Three replicates were set up for each group, and appropriate gel concentrations were selected. The electrophoresis conditions were as follows: 80 V for 22 min for the stacking gel and 120 V for 90 min for the resolving gel. A transfer membrane “sandwich” was prepared, and proteins were transferred to a PVDF membrane using the wet transfer method. The membrane was blocked with 5% nonfat milk and incubated with primary antibodies against TGF‐*β*1 (1:1000), Smad2 (1:1000), Smad3 (1:1000), and GAPDH (1:2000) at 4°C overnight. On the second day, after washing with TBST on a shaking bed, the membrane was incubated with a secondary antibody (1:10,000) for 1 h. Following another wash with TBST, ECL luminescent solution was added, and the membrane was developed on a machine.

### 2.14. Immunofluorescence (IF)

Cells from each group were seeded into confocal dishes. After intervention, the culture medium was aspirated and discarded, and the cells were washed three times with PBS. The cells were then fixed with 4% cell tissue fixative for 15 min, followed by three washes with PBS. Next, 0.2% Triton X‐100 (100 *μ*L) was added for membrane permeabilization, and the cells were incubated at room temperature for 20 min. After another three washes with PBS, the cells were blocked with 5% BSA for 40 min. Subsequently, the cells were incubated with TGF‐*β*1 (1:200) at 4°C overnight. On the second day, the cells were incubated with the secondary antibody for 1 h in the dark, and the nuclei were stained with DAPI. Finally, the cells were observed under a fluorescence microscope.

### 2.15. Statistical Analysis

Statistical analysis was performed using SPSS 20.0. Results were expressed as mean ± standard deviation. Comparison among multiple groups was conducted using one‐way ANOVA, and *p* values < 0.05 were considered statistically significant. When comparing two groups, the normal distribution of each group was tested. An independent *t*‐test was performed if both groups met normality criteria; otherwise, nonparametric tests were used. Again, a *p* value less than 0.05 was regarded as statistically significant. Bar graphs were generated using GraphPad Prism 9.

## 3. Results

### 3.1. UPLC‐MS/MS Analysis of YXF‐Containing Serum

Figure 1 illustrates the total ion chromatogram (TIC), which visually demonstrates the ion signal intensity of metabolites at different retention times, enabling qualitative and semiquantitative analysis of the samples. In the multiple reaction monitoring (MRM) chromatogram, peaks of distinct colors represent different metabolites, thereby significantly enhancing selectivity and sensitivity. Statistical analysis of metabolite classification revealed that the primary constituents in YXF‐containing serum were alkaloids (30.02%), terpenoids (25.3%), and flavonoids (11.97%). Collectively, these three categories accounted for over 67% of the total metabolites, suggesting their potential role as the key pharmacologically active components underlying the therapeutic effects of YXF.

Figure 1Metabolite analysis results of YXF‐containing serum. (a) TIC in negative ion mode. (b) TIC in positive ion mode. (c) MRM metabolite detection chromatogram in negative ion mode. (d) MRM metabolite detection chromatogram in positive ion mode. (e) Ring chart of metabolite category distribution.

**Figure:**
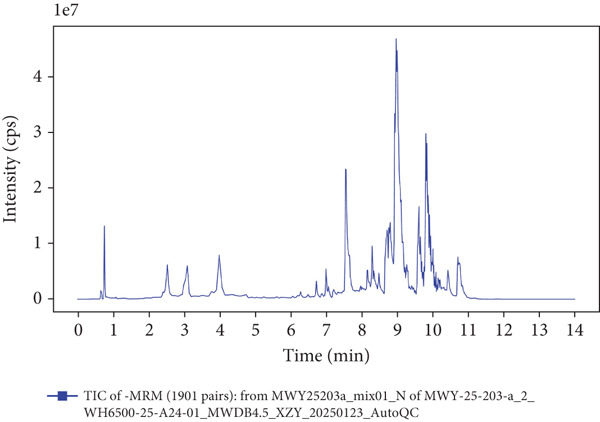
(a)

**Figure:**
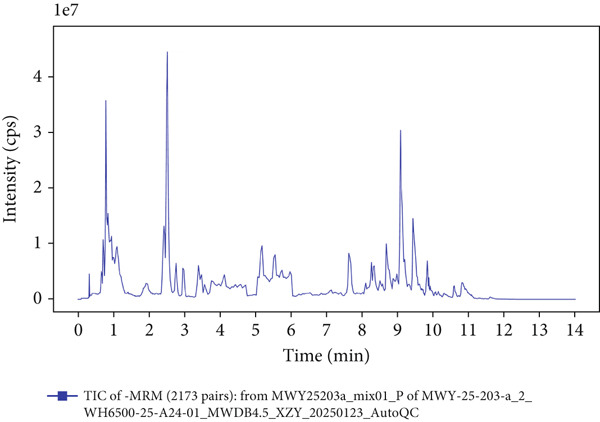
(b)

**Figure:**
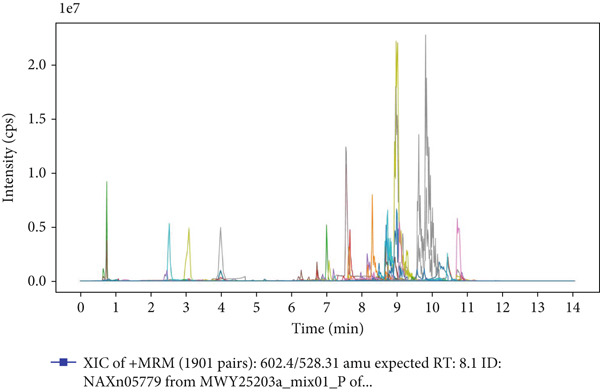
(c)

**Figure:**
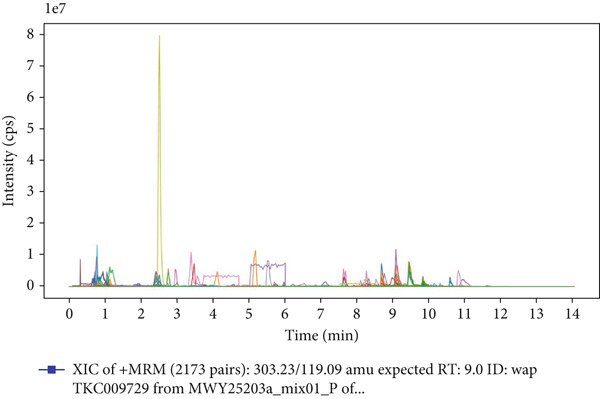
(d)

**Figure:**
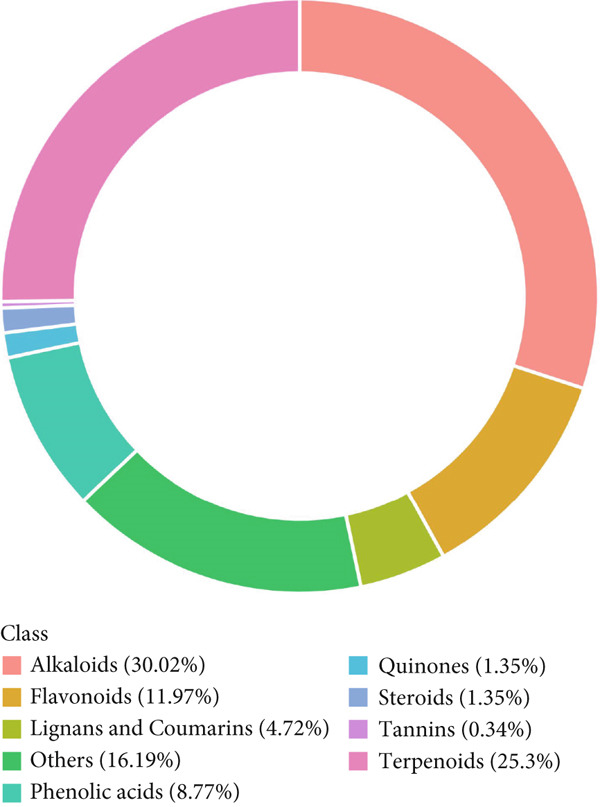
(e)

### 3.2. YXF Reduced FBG and Improved Cardiac Diastolic Function

As shown in Figure 2a,b, the body weight and FBG levels in the DCM group were significantly higher than those in the control group (*p* < 0.01). YXF treatment reduced FBG levels in DCM mice from the eighth week onwards (*p* < 0.01). In contrast, the losartan group did not demonstrate a reduction in FBG (*p* > 0.05). To further investigate the effects of YXF on glucose tolerance and insulin sensitivity in diabetic mice, OGTT measurements and AUC calculations were performed.

Figure 2YXF improves glucose metabolism and benefits diastolic function in diabetic mice. (a) Changes in body weight of mice in each group. (b) Fasting blood glucose levels in mice of each group. (c) OGTT results for mice in each group. (d) AUC area for mice in each group. (e) Changes in LVEF for mice in each group. (f) Changes in *E*/*e* ^′^ for mice in each group. Quantitative analysis of *E*/*e* ^′^. (g) Pulse wave Doppler. (h) Tissue Doppler.  ^∗^ *p* < 0.05 or  ^∗∗^ *p* < 0.01 vs. the model group; ^△^ *p* < 0.05 or ^△△^ *p* < 0.01 vs. the DCM + losartan group.

**Figure:**
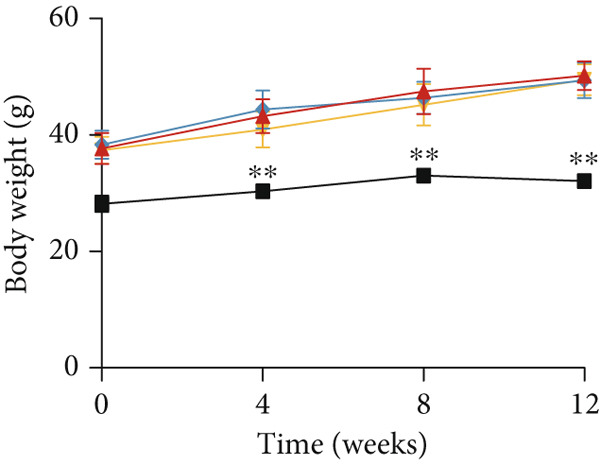
(a)

**Figure:**
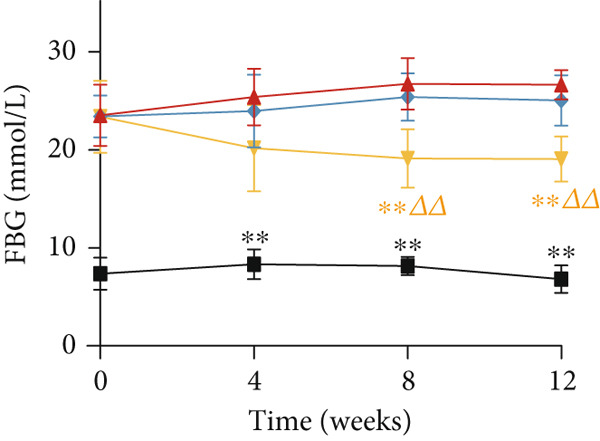
(b)

**Figure:**
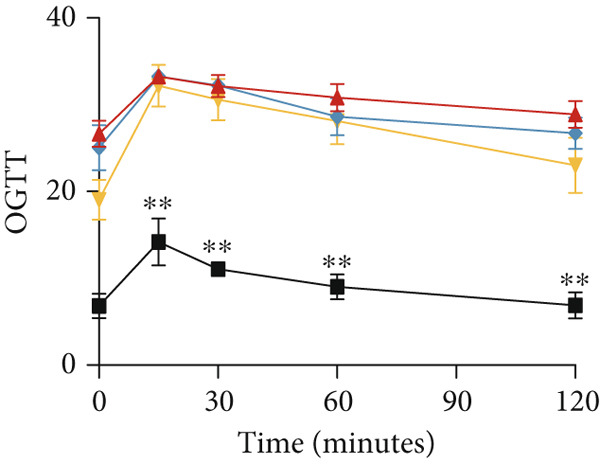
(c)

**Figure:**
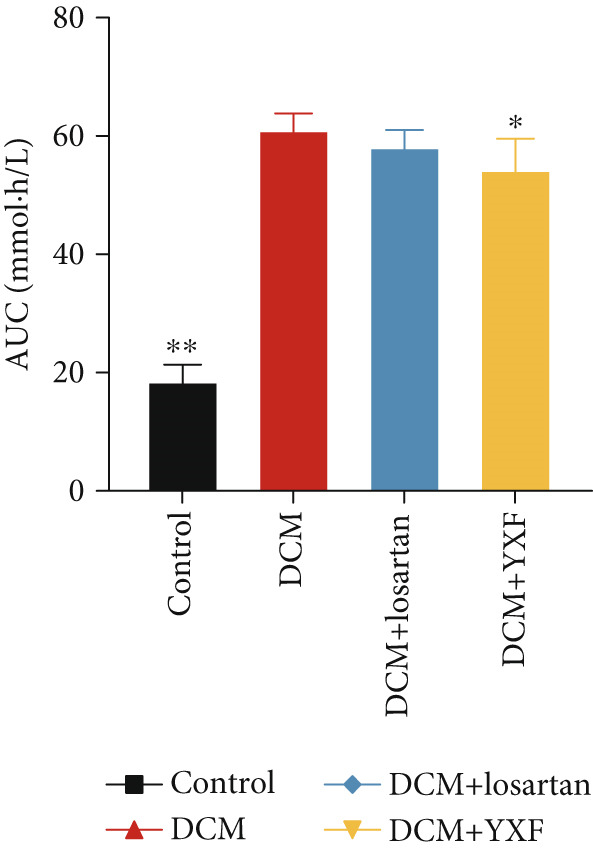
(d)

**Figure:**
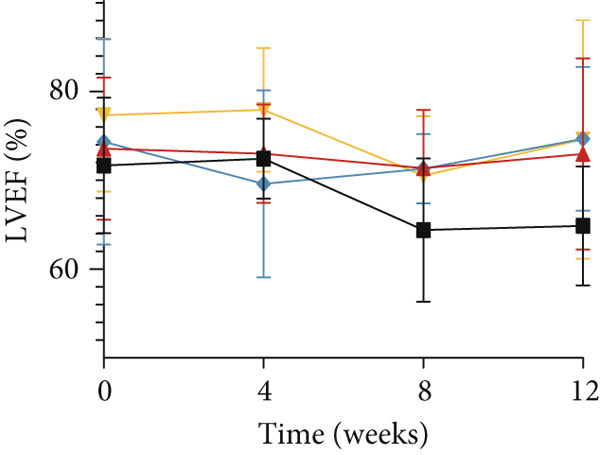
(e)

**Figure:**
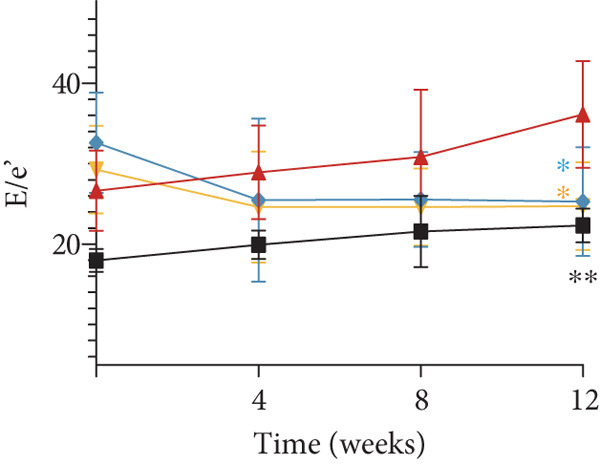
(f)

**Figure:**
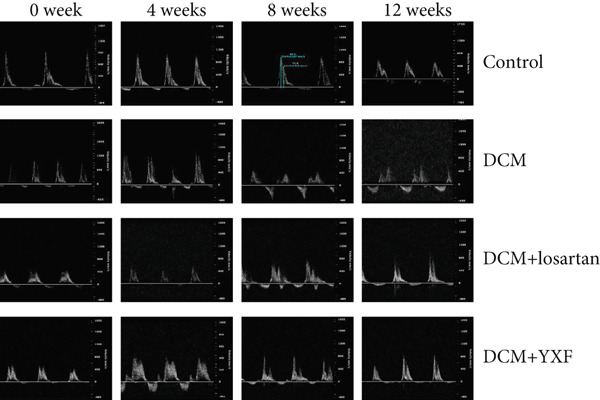
(g)

**Figure:**
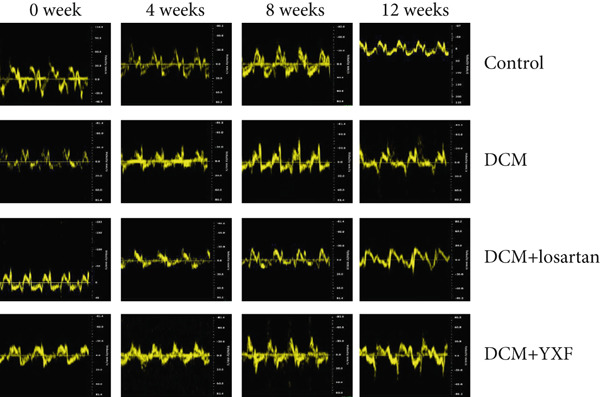
(h)

As indicated in Figure 2c,d, blood glucose levels at 0, 30, 60, and 120 min were higher in the DCM group compared to the control group (*p* < 0.01). Correspondingly, the AUC for OGTT was significantly increased by 3.265 times (Figure 2c; *p* < 0.01). After 12 weeks of YXF intervention, AUC decreased, with notable reductions in blood glucose levels at 0 and 120 min. To further elucidate the impact of YXF on cardiac function in diabetic mice, echocardiography was performed to assess LVEF% as an indicator of systolic function and the *E*/*e* ^′^ ratio as a marker of diastolic function.

As presented in Figures 2e, 2f, 2g, and 2h, compared to the control group, there was no significant difference in LVEF values in the model group (*p* > 0.05). At the same time, the *E*/*e* ^′^ ratio was significantly increased in the model group (*p* < 0.05). Over time, the *E*/*e* ^′^ ratio gradually rose in the model group mice. However, after 4 weeks of intervention with YXF and losartan, the *E*/*e* ^′^ ratio decreased and stabilized.

### 3.3. Inhibition of Myocardial Fibrosis in Diabetic Mice by YXF

According to Figure 3. The model group showed significantly higher LVAWT and LVPWT at 0 weeks of drug administration compared with the control group, while LVEDD decreased (*p* < 0.01), as shown in A‐D and M‐mode echocardiography. It suggests that the model group of mice had concentric left ventricular hypertrophy prior to drug administration. With increasing age, the LVAWT and LVPWT of the model group mice gradually thickened, suggesting a sustained effect of T2DM on left ventricular wall thickness. At the 8th and 12th weeks of drug administration, the LVAWT and LVPWT in the YXF and losartan groups were significantly reduced compared with the model group (*p* < 0.05). At the 12th week of administration, there was also a significant difference in LVEDD levels between the YXF group, the losartan group, and the model group (*p* < 0.05).

Figure 3YXF inhibits myocardial fibrosis in diabetic mice. (a) M‐mode echocardiography of the heart in mice from each group at 0, 4, 8, and 12 weeks of intervention. (b) LVAWT status in mice from each group. (c) LVEDD status in mice from each group. (d) LVPWT area in mice from each group. (e) Masson’s trichrome staining of myocardial interstitium and blood vessels in mice from each group after 12 weeks of intervention. (f) Relative area of myocardial interstitial fibrosis in mice from each group. (g) Relative area of perivascular fibrosis in mice from each group.  ^∗^ *p* < 0.05 or  ^∗∗^ *p* < 0.01 vs. the model group.

**Figure:**
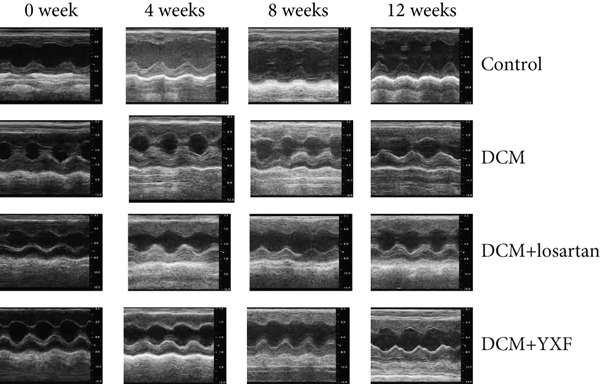
(a)

**Figure:**
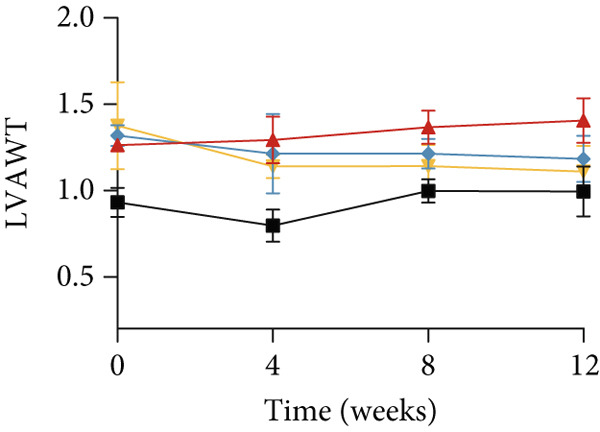
(b)

**Figure:**
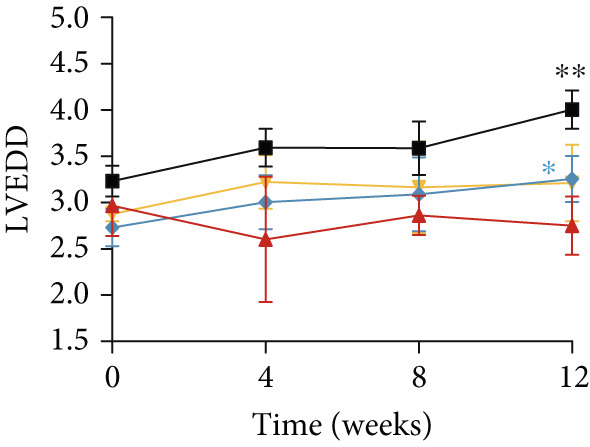
(c)

**Figure:**
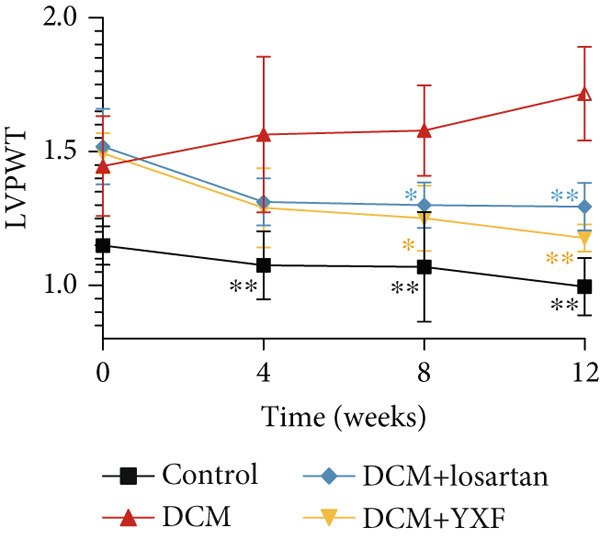
(d)

**Figure:**
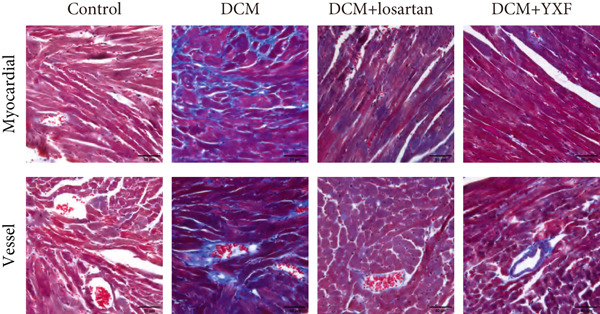
(e)

**Figure:**
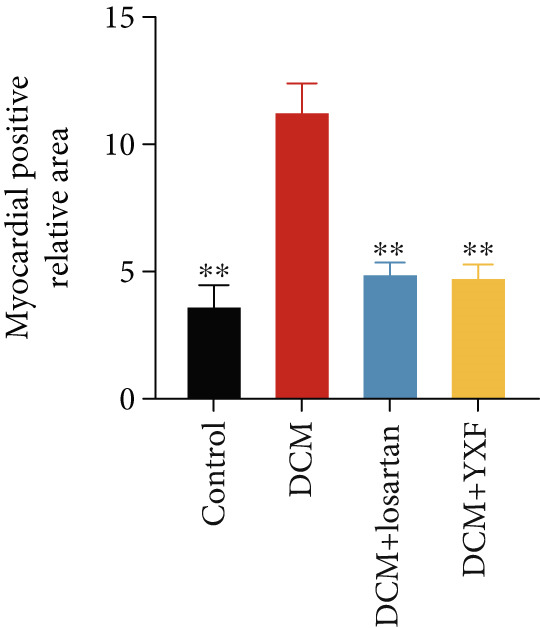
(f)

**Figure:**
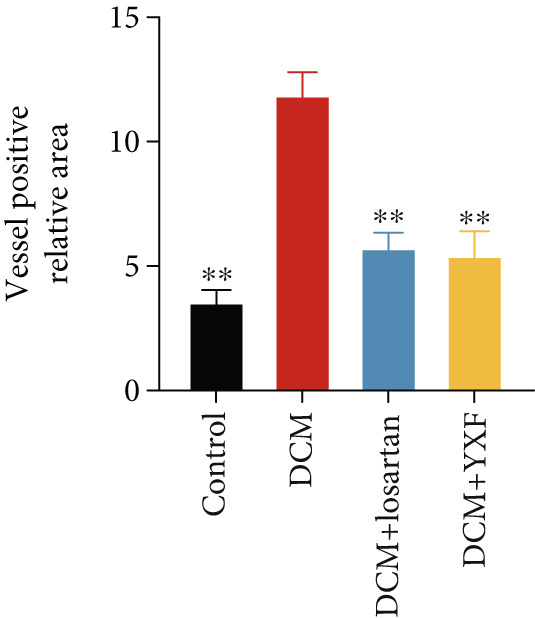
(g)

To assess the degree of myocardial fibrosis, Masson staining was performed around myocardial interstitial and perivascular areas, and fibrotic areas were measured (Figures 3e, 3f, and 3g). The results showed a significant increase in fibrotic areas in the model group compared to the control group. After 12 weeks of drug administration, the fibrotic areas around the myocardial interstitial and perivascular regions in the YXF group and the losartan group were significantly lower than those in the model group (*p* < 0.01).

### 3.4. YXF Can Inhibit the TGF‐*β*/Smads Pathway and Increase miR133a Expression

To observe the effects of YXF on myocardial fibrosis, we examined the distribution of Collagen 1 and Collagen 3 in the myocardium of mice from each group at 12 weeks of administration. As shown in Figure 4a, compared with the control group, both COL‐1 and COL‐3 were significantly increased in the model group, while they decreased after 12 weeks of YXF intervention.

Figure 4YXF can inhibit the TGF‐*β*/Smads pathway and increase the expression of miR133a. (a) Immunohistochemistry of COL‐1 and COL‐3 in mice from each group after 12 weeks of intervention. (b) Western blot images of TGF‐*β*, Smad2, and Smad3 in mice from each group after 12 weeks of intervention, along with quantitative analysis of (c) TGF‐*β*, (d) Smad2, and (e) Smad3. Quantitative analysis of mRNA levels of (f) TGF‐*β*, (g) Smad2, and (h) Smad3. (i) RNA measurement of miR133a in the myocardial tissue of mice from each group after 12 weeks.  ^∗^ *p* < 0.05 or  ^∗∗^ *p* < 0.01 vs. the model group.

**Figure:**
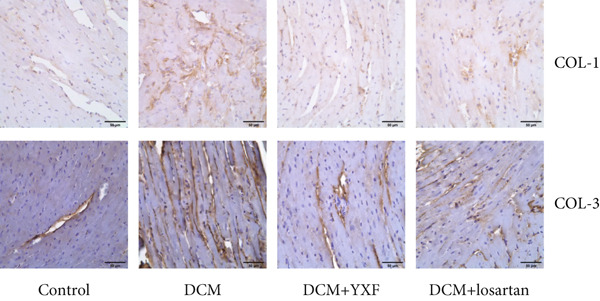
(a)

**Figure:**
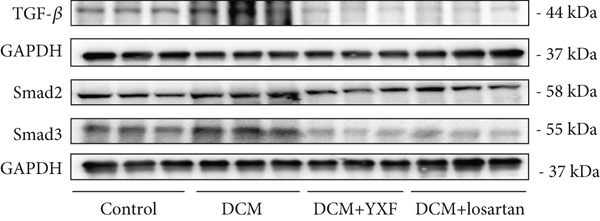
(b)

**Figure:**
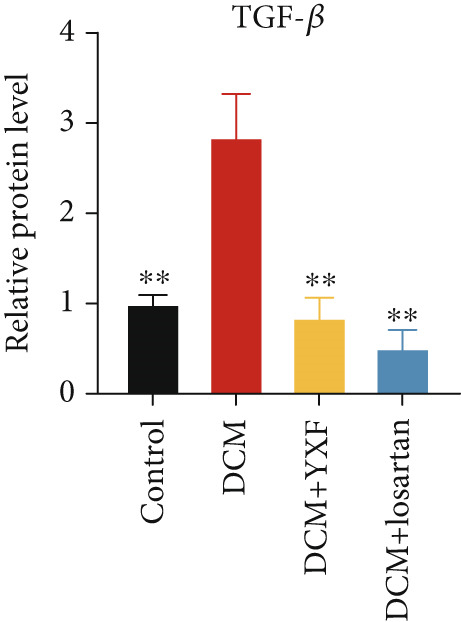
(c)

**Figure:**
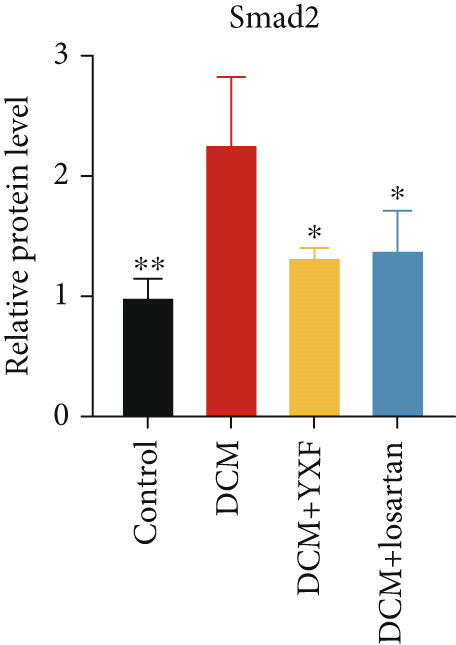
(d)

**Figure:**
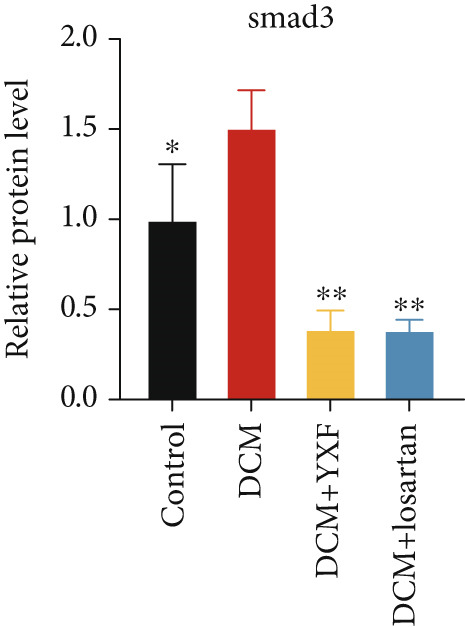
(e)

**Figure:**
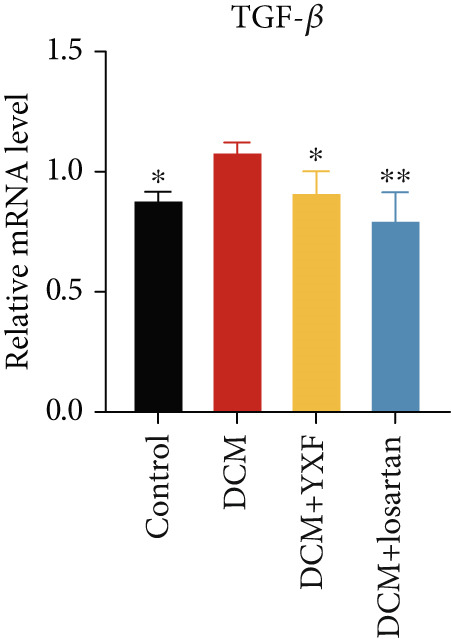
(f)

**Figure:**
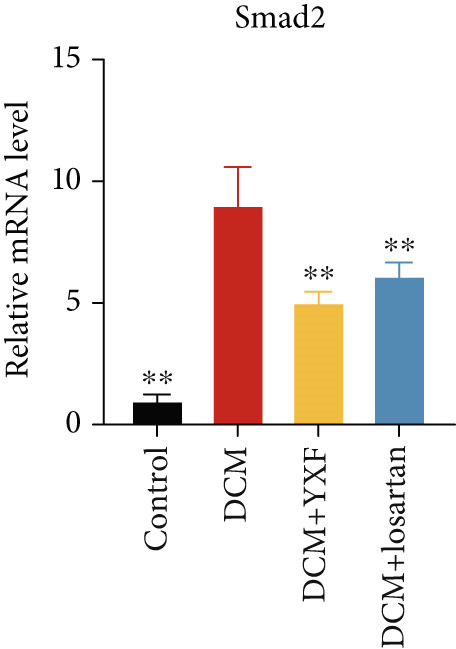
(g)

**Figure:**
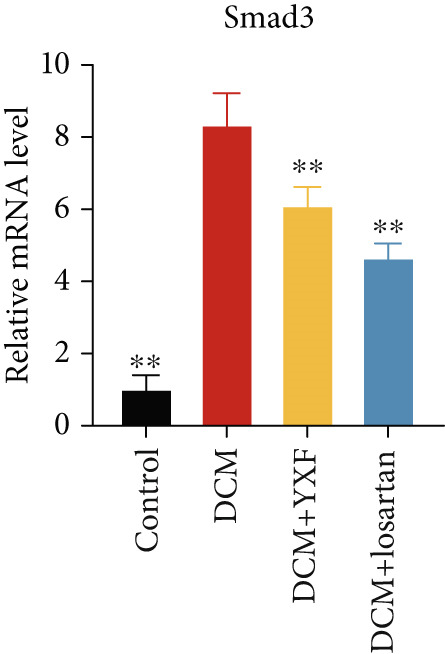
(h)

**Figure:**
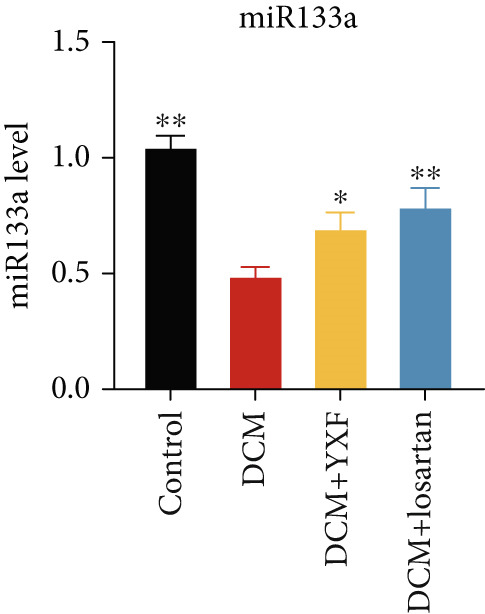
(i)

Due to technical reasons, we were unable to obtain reliable protein data for COL‐1 and COL‐3 in our in‐cell experiments. As an alternative, we assessed periostin expression, a key upstream regulator that drives fibroblast differentiation and COL‐1 and COL‐3 synthesis [14]. As shown in Figure S1, YXF inhibited the upregulation of periostin in the model group. This result aligns with our central findings.

As expected, compared with the control group, the expression of TGF‐*β*, Smad2, and Smad3 in the myocardial tissue of mice in the model group was significantly increased (Figures 4b, 4c, 4d, and 4e). After 12 weeks of YXF intervention, the expression of TGF‐*β*, Smad2, and Smad3 decreased (*p* < 0.05). Consistent with this, as shown in Figures 4f, 4g, and 4h, there were significant differences in TGF‐*β*, Smad2, and Smad3 mRNA levels between the YXF intervention group and the model group after 12 weeks (*p* < 0.05).

Studies have shown that miR‐133a expression decreases in patients with DCM [15]. We examined miR‐133a levels in myocardial tissue and found that miR‐133a expression was significantly decreased in the model group (*p* < 0.05), consistent with previous reports. However, YXF could restore miR‐133a expression (Figure 4i).

### 3.5. YXF Inhibited the TGF‐*β*/Smads Pathway in MCFs

Next, we first investigated the expression of the TGF‐*β*/Smads pathway in MCFs and explored the effects of YXF‐mediated serum on this pathway. As shown in Figure 5a, the mRNA expression of TGF‐*β*, Smad2, and Smad3 was increased in the model group of MCFs, which was consistent with the animal experiments. Figure 5b,c demonstrates that YXF treatment significantly inhibited the protein expression of TGF‐*β*, Smad2, and Smad3 in high‐glucose‐cultured cardiac fibroblasts (*p* < 0.05). Additionally, Figure 5d shows that TGF‐*β* was localized in the cytoplasm of MCFs, with higher expression in the model group than in the control group. However, TGF‐*β* expression decreased after YXF treatment.

Figure 5YXF‐medicated serum inhibits the TGF‐*β*/Smads pathway in mouse cardiac fibroblasts. (a) Quantitative analysis of mRNA levels of TGF‐*β*, Smad2, and Smad3 in mouse cardiac fibroblasts from each group. (b) Western blot images of TGF‐*β*, Smad2, and Smad3 in mouse cardiac fibroblasts from each group, along with (c) quantitative analysis. (d) Immunofluorescence images of TGF‐*β* in mouse cardiac fibroblasts from each group.  ^∗^ *p* < 0.05 or  ^∗∗^ *p* < 0.01 vs. the model group.

**Figure:**
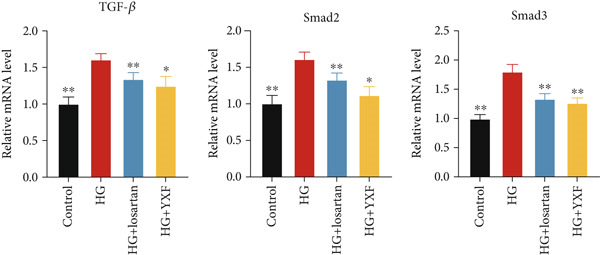
(a)

**Figure:**
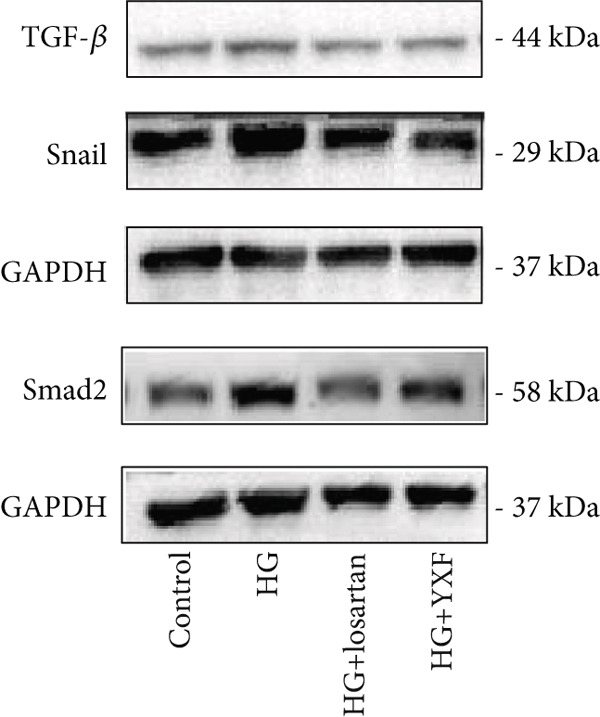
(b)

**Figure:**
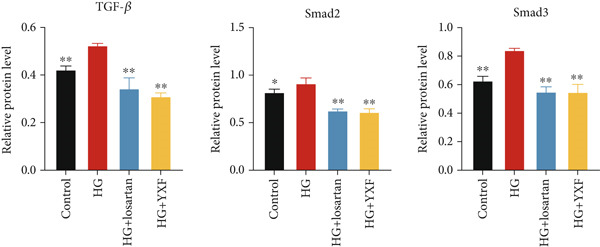
(c)

**Figure:**
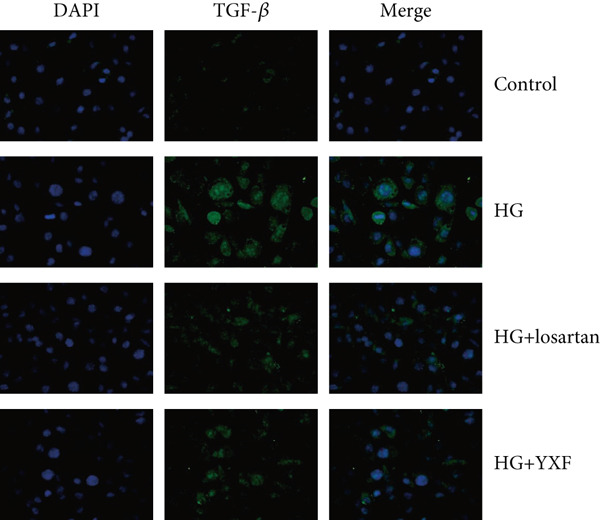
(d)

### 3.6. Suppression of miR133a Reduces the Therapeutic Effect of YXF

To further investigate the relationship between YXF and miR133a, we transfected MCFs in a high‐glucose environment with a miR133a inhibitor, in addition to YXF intervention (Figure 6a). The results indicated that inhibiting miR‐133a increased mRNA expression of TGF‐*β*, Smad2, and Smad3 (*p* < 0.05). As shown in Figure 6c, the IF of TGF‐*β* and Smad3 was significantly increased in the miR133a inhibitor group. Furthermore, as demonstrated in Figure 6d,e, the protein expression of TGF‐*β*, Smad2, and Smad3 also increased upon miR133a inhibition. Taken together, these findings strongly suggest that inhibiting miR133a can reduce the therapeutic effect of YXF and activate the TGF‐*β*/Smads pathway.

Figure 6The reduction in YXF′s therapeutic effect after miR133a inhibition. (a) The miR133a expression levels in the control group and the miR133a inhibitor group were both receiving YXF intervention. (b) The quantitative analysis of mRNA for TGF‐*β*, Smad2, and Smad3 in cells from the control and miR133a inhibitor groups. (c) Immunofluorescence images of TGF‐*β* and Smad3 in cells from both groups. (d, e) Western blot images and quantitative analysis of TGF‐*β*, Smad2, and Smad3 protein expression in cells from the control and miR133a inhibitor groups.  ^∗^ *p* < 0.05 or  ^∗∗^ *p* < 0.01 vs. the miR133a NC group.

**Figure:**
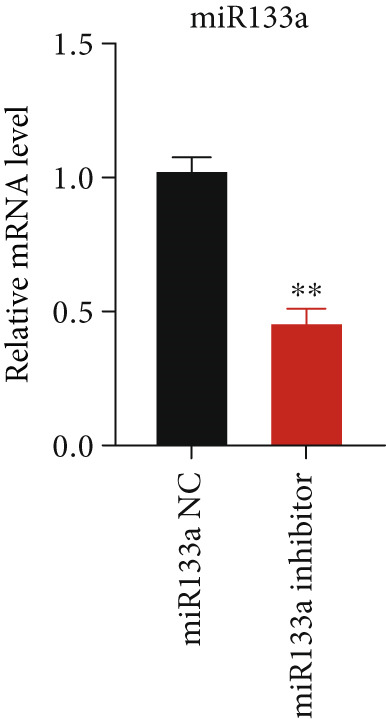
(a)

**Figure:**
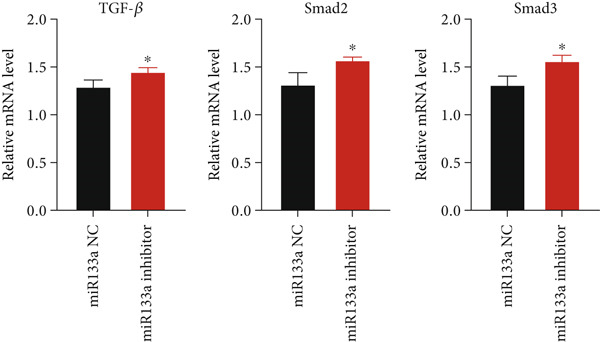
(b)

**Figure:**
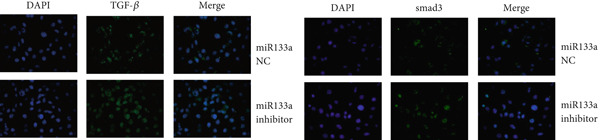
(c)

**Figure:**
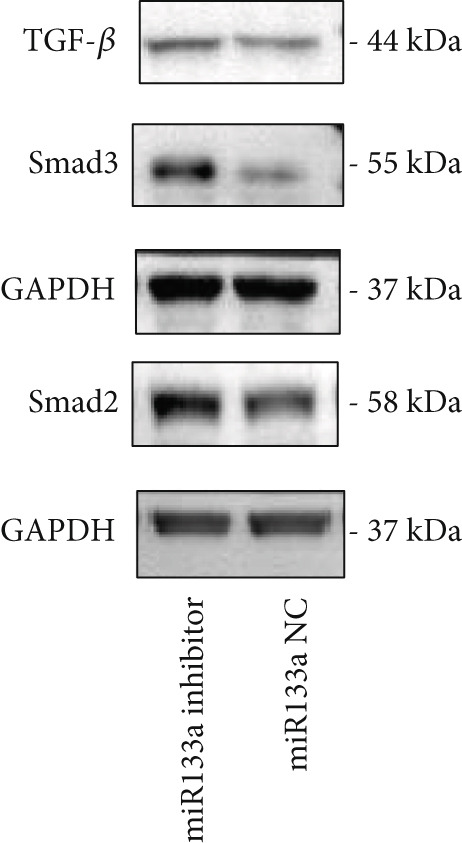
(d)

**Figure:**
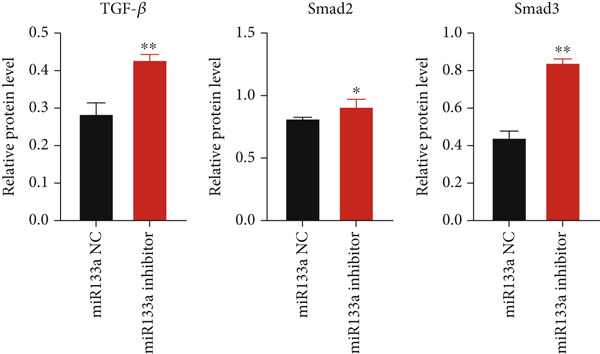
(e)

## 4. Discussion

DCM is a disease characterized by structural and functional heart changes caused by diabetes. Currently, the widely used DCM model is the inducible animal model [16], but this model has an unstable success rate and requires exploration of injection methods and dosages. The spontaneous Type 2 diabetes KKAy mouse, with its inherent insulin resistance, has been widely used as an animal model of obesity and mild hyperglycemia to explore the pathogenesis of human Type 2 diabetes [17]. Myocardial fibrosis in DCM manifests microscopically as excessive proliferation of MFs around interstitial and intramyocardial blood vessels. As fibrosis increases, it leads to cardiac structural remodeling and diastolic dysfunction [18]. In this study, through cardiac function testing and pathological observation of spontaneous diabetic KKAy mice, it was found that their blood glucose levels continued to rise after being fed a high‐fat diet, with a steady increase in ventricular wall thickness, accompanied by persistent myocardial fibrosis and diastolic dysfunction. Pathological examination showed a disordered arrangement of cardiomyocytes and increased intercellular spaces, consistent with the features of DCM.

TCM is considered one of the primary alternative medical forms for treating DCM. In YCM, DCM belongs to the stage of consumptive thirst. As mentioned in “Ling Shu: Wu Bian”, “Those with weak five organs are prone to consumptive thirst.” It indicates that the heart disease of consumptive thirst is mainly due to deficiency, with both qi and yin deficient in the heart, leading to the generation of pathological factors such as phlegm and blood stasis. The disease involves the heart and is related to the liver, kidneys, and spleen. Based on ancient literature and clinical practice, YXF is centered on the theory of “microsyndromes of heart meridians,” with the treatment principle of nourishing qi and yin, and eliminating syndromes and scattering knots. In the formula, a large amount of *Astragalus membranaceus* is used to nourish qi and yin, promoting qi and blood circulation. *Panax notoginseng* and *Scolopendra* directly enter the blood meridians, clearing blood stasis and unblocking meridians. *Prunella vulgaris* and *Euonymus alatus* dispel stagnant qi and syndromes. Existing research shows that *Astragalus membranaceus* can improve insulin sensitivity, protect islet cells, and modulate intestinal flora, thereby exerting a hypoglycemic effect [19, 20]. Salvianolic acids, as extracts of *Salvia miltiorrhiza*, and 1‐ethyl‐3‐formyl‐*β*‐carbolines, as extracts of Radix Pseudostellariae, both have cardioprotective effects [21, 22]. *Ophiopogon japonicus* polysaccharides, one of the main active ingredients of *Ophiopogon japonicus*, have biological activities such as hypoglycemic, cardioprotective, and obesity‐improving effects [23]. Studies have shown that *Schisandra chinensis* fruit extract (SCE) and schisandrin B (SchB) can inhibit the TGF‐*β* signaling pathway in vascular smooth muscle cells [24]. Extracts of *Angelica sinensis*, *Paeonia lactiflora*, and *Prunella vulgaris* exhibit pharmacological effects, including anti‐inflammatory and antioxidant activities [25–27]. Currently, there is less research on *Euonymus alatus* and *Fritillaria thunbergii*, but in this formula, they play a role in regulating qi and dispersing knots.

Our pharmacological studies have shown that YXF can significantly reduce the *E*/*e* ^′^ value in DCM mice while improving LVAWT and LVPWT. In addition, the myocardial cells of YXF‐treated mice are more neatly arranged and tightly packed, with less collagen between cells. In other words, YXF is beneficial for restoring ventricular function and delaying pathological remodeling.

The TGF‐*β*/Smads pathway is a classical pathway for fibrosis. The TGF‐*β* family is large and diverse, with TGF‐*β*1 being a typical fibrogenic factor, and Smad2 and Smad3 are two major downstream regulatory factors that mediate fibrosis [28]. In the context of DCM, hyperglycemia can directly stimulate TGF‐*β*1 expression, leading to phosphorylation of Smad2 and Smad3 in the cytoplasm, which then translocate to the nucleus to induce transcription of fibrosis‐promoting genes, thereby participating in myocardial fibrosis [29]. As a classic AngII receptor antagonist, losartan inhibits the TGF‐*β* signaling pathway in cardiac fibroblasts in vitro and attenuates myocardial hypertrophy, fibrosis, and left ventricular remodeling in vivo [30–32]. Using losartan as a positive control is a common strategy in related studies of DCM and myocardial fibrosis [33].

This study shows that YXF has a similar effect on heart function as losartan, but is superior to losartan in blood glucose and weight control. This multiple effect of YXF may be related to the reasonable combination of its various chemical components.

With advances in molecular biology, noncoding RNAs are increasingly playing an important role in pathological mechanisms. The miR‐133 family consists of miR‐133a and miR‐133b, where miR‐133a is present in the myocardium, while miR‐133b is generally believed to only exist in skeletal muscle. miR‐133a has been confirmed as an important miRNA in the heart, regulating cardiac reprogramming [34]. Under high‐glucose conditions, miR‐133a is downregulated in hypertrophic myocardium, and its overexpression can reduce hypertrophic changes [35]. The results of this study indicate that under high‐glucose conditions, miR‐133a expression is upregulated in MFs, and cell viability increases, consistent with previous studies [36]. It has been verified that knocking down miR‐133a reduces the efficacy of YXF, suggesting that miR‐133a is a potentially important target for YXF. Losartan, used as a positive control in this study, also inhibits myocardial fibrosis, consistent with previous studies [37]. Compared with losartan, YXF exhibits comparable therapeutic efficacy while offering superior glycemic control.

Our study conducted a preliminary investigation into the efficacy and mechanism of YXF in treating DCM. Firstly, YXF can lower blood glucose levels and delay the onset of diastolic dysfunction in diabetic mice. Secondly, YXF alleviates myocardial fibrosis in diabetic mice by inhibiting the TGF‐*β*/Smads pathway, which may be related to its ability to increase myocardial miR133a levels. However, this study has certain limitations. The direct targeting relationship between miR133a and TGF‐*β* remains to be further verified, and the practical components of YXF that improve myocardial fibrosis in diabetic mice need to be further identified. Additionally, this study focused only on the effects of YXF on myocardial fibrosis, while other aspects, such as inflammation and oxidative stress, remain to be investigated.

## 5. Conclusion

In this study, YXF can upregulate miR133a, inhibit the myocardial TGF‐*β*/Smads pathway, improve blood glucose levels and myocardial fibrosis, and thus have the potential to treat DCM. In future research, we plan to perform in vivo causal validation through pathway blockade, which will help establish a more precise experimental foundation for developing and applying these herbal medicines in DCM treatment.

## Conflicts of Interest

The authors declare no conflicts of interest.

## Author Contributions

**Qiyao Zhao:** writing – original draft and the cell experiment part of the work. **Honghui Wu and Yalu Wen:** pathology of animal experiments. **Jiaoyue Li and Yunpeng Luo:** the experimental work involving cells. **Ping Li, Ye Zhang, and Chaoyue Hu**: Animal breeding**. Jukai Huang, Li Zhang, and Xiaohui Yang:** writing – editing, supervision, funding acquisition. **Qiyao Zhao, Yalu Wen, Honghui Wu, and Jiaoyue Li** are co‐authors who contributed equally to this manuscript.

## Funding

This study was supported by the National Natural Science Foundation of China, 10.13039/501100001809, 81974541 and 82174192; Beijing Natural Science Foundation, 7212176; and Beijing University of Chinese Medicine Dongzhimen Hospital 2024 Annual Scientific Innovation Project, DZMKJCX‐2024‐029.

## Supporting information


**Supporting Information** Additional supporting information can be found online in the Supporting Information section. To provide complementary evidence for the antifibrotic effect of YXF, we analyzed the expression of periostin—a key upstream regulator driving collagen production. Figure S1: YXF treatment inhibited the upregulation of periostin in both in vivo and in vitro models, supporting its antifibrotic effect.

## Data Availability

The data that support the findings of this study are available on request from the corresponding author. The data are not publicly available due to privacy or ethical restrictions.
