# Supplementary material for: Yixiao Formula Suppresses Myocardial Fibrosis Through UpregulatingmiR‐133a and Downregulating TGF‐β/Smads Signal Molecules
Source: J Diabetes Res. 2026 Jan 21;2026:5533249. doi: 10.1155/jdr/5533249 (PMC12822571; doi:10.1155/jdr/5533249)
Supplement: Supplementary file 1 — Supporting Information Additional supporting information can be found online in the Supporting Information section. To provide complementary evidence for the antifibrotic effect of YXF, we analyzed the expression of periostin—a key upstream regulator driving collagen production. Figure S1: YXF treatment inhibited the upregulation of periostin in both in vivo and in vitro models, supporting its antifibrotic effect. [file JDR-2026-5533249-s001.docx]

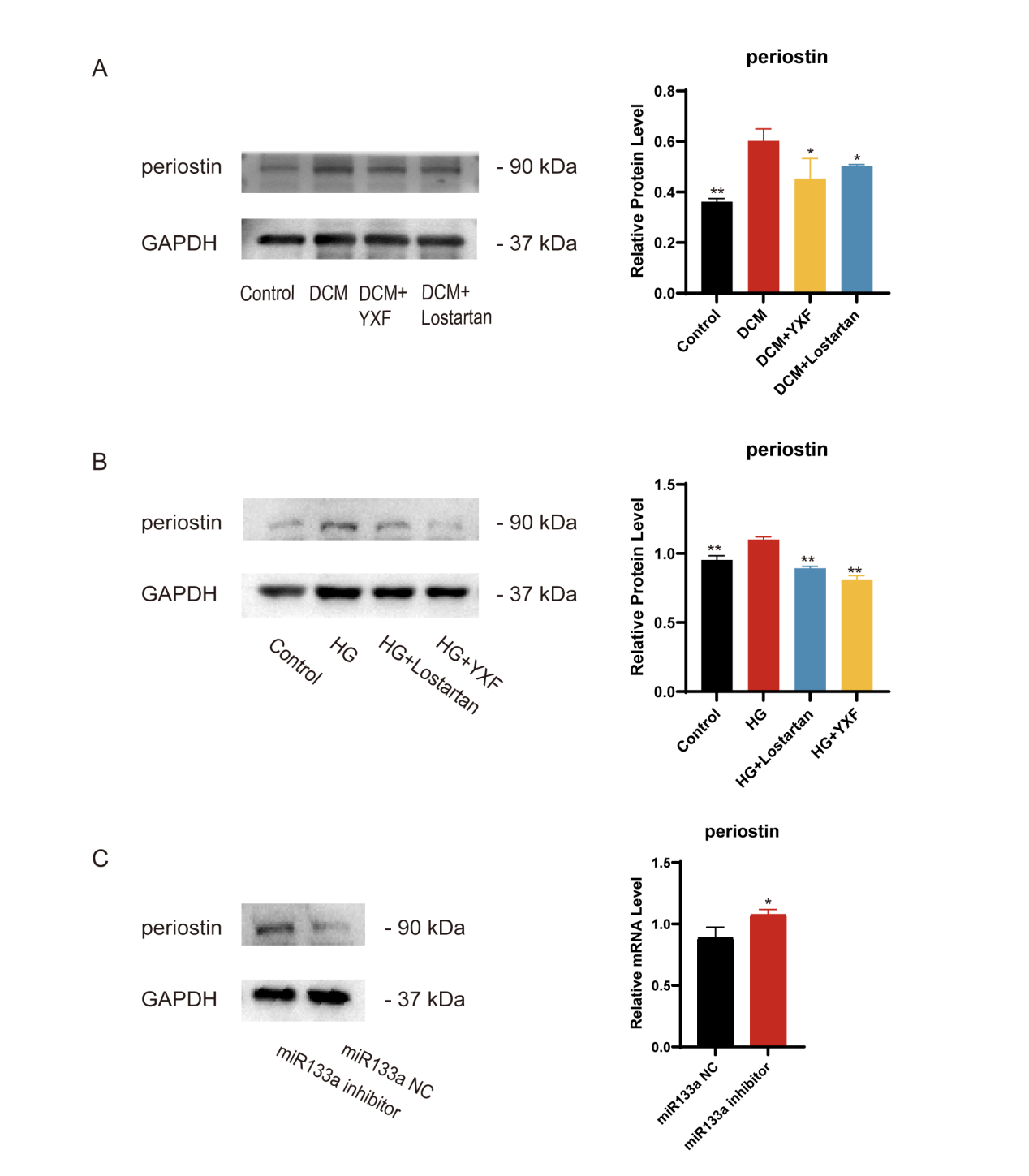


**Fig.S1** the expression level of Periostin in vivo and in vitro. Western blot images of periostin in mice (A), mouse cardiac fibroblasts (B) and in cells from the control and miR133a inhibitor groups (C) along with quantitative analysis.
